# The pan-Bcl-2 inhibitor obatoclax promotes differentiation and apoptosis of acute myeloid leukemia cells

**DOI:** 10.1007/s10637-020-00931-4

**Published:** 2020-05-04

**Authors:** Małgorzata Opydo-Chanek, Iwona Cichoń, Agnieszka Rak, Elżbieta Kołaczkowska, Lidia Mazur

**Affiliations:** 1 Department of Experimental Hematology, Institute of Zoology and Biomedical Research, Jagiellonian University, Gronostajowa 9, 30-387 Kraków, Poland; 2 Department of Physiology and Toxicology of Reproduction, Institute of Zoology and Biomedical Research, Jagiellonian University, Gronostajowa 9, 30-387 Kraków, Poland

**Keywords:** Obatoclax, Differentiation, Apoptosis, Acute myeloid leukemia, HL-60 cells

## Abstract

One of the key features of acute myeloid leukemia (AML) is the arrest of differentiation at the early progenitor stage of myelopoiesis. Therefore, the identification of new agents that could overcome this differentiation block and force leukemic cells to enter the apoptotic pathway is essential for the development of new treatment strategies in AML. Regarding this, herein we report the pro-differentiation activity of the pan-Bcl-2 inhibitor, obatoclax. Obatoclax promoted differentiation of human AML HL-60 cells and triggered their apoptosis in a dose- and time-dependent manner. Importantly, obatoclax-induced apoptosis was associated with leukemic cell differentiation. Moreover, decreased expression of Bcl-2 protein was observed in obatoclax-treated HL-60 cells. Furthermore, differentiation of these cells was accompanied by the loss of their proliferative capacity, as shown by G0/G1 cell cycle arrest. Taken together, these findings indicate that the anti-AML effects of obatoclax involve not only the induction of apoptosis but also differentiation of leukemic cells. Therefore, obatoclax represents a promising treatment for AML that warrants further exploration.

## Introduction

Acute myeloid leukemia (AML) is a hematological malignancy in which the bone marrow is replaced by a clonal population of abnormal myeloid progenitors [1]. The major feature of AML is differentiation arrest of these progenitor cells at early stages of myelopoiesis. This is further accompanied by enhanced cell proliferation and resistance to death-inducing signals. Therefore, AML is a disorder of impaired hematopoietic cell differentiation and apoptosis. Therapies aimed at resuming the process of maturation and apoptosis in leukemic cells are currently the most promising anti-AML strategies [1]. Treatment of acute promyelocytic leukemia (APL), a subtype of AML, with all-*trans*-retinoic acid (ATRA) was the first model of differentiation-targeted therapy [2]. Despite its spectacular clinical success, ATRA-based therapy has limitations due to both the induction of potentially life-threatening toxicity and the acquisition of resistance in some patients [3]. Moreover, most non-APL AML patients do not benefit from ATRA therapy. Therefore, the search for new compounds that can efficiently and selectively induce differentiation and apoptosis in leukemic cells remains essential for the future of AML therapy, especially concerning non-APL subtypes of AML.

Recently, proteins of the B cell lymphoma-2 (Bcl-2) family have attracted considerable interest as therapeutic targets in AML [4–6]. The Bcl-2 protein family is a key regulator of the mitochondrial pathway of apoptosis. Based on structural and functional features, three different subclasses of the Bcl-2 protein family are distinguished. Proteins of the anti-apoptotic Bcl-2 subfamily, such as Bcl-2, Bcl-xL and Mcl-1, inhibit apoptosis by preserving mitochondrial membrane integrity [7]. These proteins bind to and inactivate Bax and Bak, the multidomain members of the pro-apoptotic subfamily. In response to apoptotic signals, Bax and/or Bak undergo conformational changes and oligomerize, forming pores in the outer mitochondrial membrane. Mitochondrial membrane permeabilization leads to the release of death-promoting proteins and, consequently, activation of the initiator caspase 9 and effector caspases 3 and 7, resulting in the morphological and biochemical changes associated with apoptosis [8]. Proteins of the third subfamily, named “BH3-only” proteins, carry out their pro-apoptotic function through two mechanisms: neutralization of the anti-apoptotic Bcl-2 subfamily proteins and direct activation of the pro-apoptotic effectors Bax and Bak [7, 8]. The balance between anti-apoptotic and pro-apoptotic members of the Bcl-2 family, mediated through protein-protein interactions, determines cell survival or death.

Besides being important regulators of apoptosis, proteins of the Bcl-2 family also govern other cellular pathways, including the cell cycle, proliferation and differentiation [9, 10]. Several studies have shown that Bcl-2 proteins are expressed in early hematopoietic progenitors and regulated during the differentiation of myeloid cells [11]. Moreover, there is mounting evidence that the balanced regulation of Bcl-2 proteins is crucially important in the generation, maintenance and function of the hematopoietic system [12–14]. Importantly, the expression of Bcl-2 and/or other members of the anti-apoptotic subfamily is often elevated in hematological malignancies, either as a result of gene translocations or amplifications, or due to mechanisms independent from genomic aberrations [15]. The over-expression of anti-apoptotic Bcl-2 family members contributes to enhanced leukemic cell survival, chemoresistance and poor clinical outcomes, suggesting that these proteins may be an attractive therapeutic target [16–18].

Obatoclax, a synthetic indole bipyrrole derivative of bacterial prodiginines, is a small-molecule inhibitor of the anti-apoptotic proteins of the Bcl-2 family that was designed to mimic pro-apoptotic BH3-only proteins in binding to anti-apoptotic Bcl-2 family members. Obatoclax antagonizes Bcl-2, Bcl-xL, Bcl-w and Mcl-1, thus leading to the activation of Bax/Bak, mitochondrial depolarization, cytochrome c release and subsequent activation of caspase-3, which consequently leads to apoptosis in cancer cells [19, 20]. Moreover, obatoclax enhances apoptosis when combined with conventional chemotherapeutics or targeted therapy drugs, as has been shown in preclinical in vitro and in vivo studies [21, 22]. Several phase I and II clinical trials have been completed investigating the use of obatoclax as a single agent in the treatment of solid tumors and hematological malignancies, including AML, chronic lymphocytic leukemia, acute lymphoblastic leukemia, myelodysplastic syndrome and Hodgkin’s lymphoma, however, all of these trials demonstrated only modest efficacy [23]. Additionally, evidence from clinical trials has indicated that obatoclax may be more effective when used in combination with other anti-cancer therapeutics, however further studies are required to fully investigate this issue [23, 24]. Despite the clinical developments regarding obatoclax, its anti-cancer activity is still not fully understood. Recent investigations have highlighted additional mechanisms behind obatoclax’s anti-cancer actions, including the inhibition of proliferation and migration of colorectal [25] and esophageal [26] cancer cells. Initial studies have shown that obatoclax is capable of exerting anti-AML effects through the induction of cell cycle arrest and inhibition of leukemic cell growth [19, 27, 28]. Further elucidation of obatoclax’s mechanism of action may provide important data in regard to improving its therapeutic applications.

To our knowledge, thus far, the pro-differentiating effect of obatoclax on AML cells has not been investigated. Therefore, the present study was designed to determine the anti-AML efficacy of obatoclax by examining its influence on differentiation and apoptosis in human AML HL-60 cells. The HL-60 cell line has been widely applied in studies on hematopoietic cell proliferation, differentiation and death [29, 30]. This cell line was derived from a 36-year-old woman who was initially considered to have APL, although subsequent evaluation has indicated that the leukemia from which HL-60 cells were derived is more appropriately classified as AML with maturation [29]. HL-60 cell cultures comprise maturation-arrested cells with properties similar to those of myeloblasts and promyelocytes, such as their characteristic large, rounded nuclei containing two to four distinct nucleoli, fine chromatin and a basophilic cytoplasm with azurophilic granules [29]. These cells can be induced to differentiate in vitro into cells of diverse myeloid lineages, depending on which reagents are used. Agents such as ATRA and dimethyl sulfoxide have been reported to induce granulocytic differentiation of HL-60 cells, whereas 12-O-tetradecanoylphorbol-13-acetate and vitamin D3 have been shown to induce their differentiation into monocytes/macrophages [30]. The course of HL-60 cell differentiation is accompanied by a number of changes in these cells that can be monitored through morphological, histochemical and immunological criteria. In addition to changes in cell morphology, the generation of reactive oxygen species, as measured by nitroblue tetrazolium (NBT)-reducing ability and expression of the αM protein subunit for the β2-integrin (CD11b), are considered specific markers of HL-60 cell differentiation [31]. It has been reported that undifferentiated HL-60 cells either do not express or only weakly express CD11b, while the expression of CD11b dramatically increases when HL-60 cells have matured to the myelocyte level [32]. Several genetic alterations of specific oncogenes have been also noted in HL-60 cells, including p53 deletion, c-myc amplification and Bcl-2 overexpression [33, 34]. In the present study, we show for the first time that obatoclax, a pan-Bcl-2 inhibitor, promotes differentiation of human AML HL-60 cells accompanied by cell cycle arrest and the down-regulation of Bcl-2 protein levels. Moreover, we demonstrate that the apoptosis of AML cells triggered by obatoclax can be differentiation-dependent.

## Material and methods

### Reagents

Obatoclax mesylate (OBAT) was purchased from Selleck Chemicals (Munich, Germany). The OBAT was dissolved in DMSO and stored as 5 mM stock solutions at −20 °C. RPMI 1640 medium and fetal calf serum were obtained from GIBCO BRL Life Technologies (Gaithersburg, MD, USA). L-glutamine, antibiotic antimycotic solution (AAS), dimethyl sulfoxide (DMSO), NBT, phorbol 12-myristate 13-acetate (PMA) and anti-β-actin antibody (cat. #A5316) were purchased from Sigma Aldrich **(**St. Louis, MO, USA). The FITC Annexin V Apoptosis Detection Kit I, propidium iodide (PI)/RNase staining buffer and anti-CD-11b were obtained from BD Biosciences (San Jose, CA, USA). The CellEvent™ Caspase 3/7 Green Flow Cytometry Assay kit was purchased from Molecular Probes (Eugene, OR, USA). The Hemacolor® Rapid Staining kit was obtained from Merck Millipore (Darmstadt, Germany). The antibody against Bcl-2 (cat. #2876) and horseradish peroxidase-conjugated secondary antibody (cat. #7074) were purchased from Cell Signaling Technology (Danvers, MA, USA).

### Cell culture and treatment

Human AML HL-60 cells were obtained from American Type Culture Collection (Rackville, USA). HL-60 cells were maintained in RPMI 1640 medium supplemented with 10% fetal calf serum, 2 mM L-glutamine and AAS containing 20 units of penicillin, 20 mg streptomycin and 0.05 mg amphotericin B. The cells were passaged every third day. The cells grew exponentially at 37 °C in an atmosphere of 5% CO_2_ in air (HERAcell incubator, Thermo Fisher Scientific). Leukemic cells were seeded in 12-, 24- or 96-well plates at a density of 1 × 10^5^ cells/mL prior to performing the experiments. HL-60 cells were treated with OBAT at a concentration of either 100 nM or 500 nM. These clinically relevant concentrations of OBAT were chosen based on previous studies [27]. The control treatments consisted of untreated and DMSO-treated HL-60 cells. The final concentration of DMSO did not influence the analyzed parameters and no significant differences in the cell response to this diluent were observed, thus the data obtained from DMSO-treated cells were considered the control.

### Cell cycle analysis

HL-60 cells were harvested by centrifugation, fixed with 70% cold ethanol at 4 °C for 60 min and then stored at −20 °C until analysis. Before staining with PI, the leukemic cell suspension was washed twice with 1 ml of PBS. After washing, the cell pellet was resuspended in 300 μl of the PI/RNase staining buffer and the cells were incubated in the dark for 30 min at room temperature. The red fluorescence of PI was measured using a FACSCalibur flow cytometer (Becton Dickinson). 10,000 cells were examined in each sample. The percentages of cell population that were in each phase of the cell cycle (G0/G1, S, G2/M), as well as the frequency of sub-G1 population, were calculated from the DNA content histograms using WinMDI 2.8 software.

### Assessment of cell differentiation

#### Analysis of leukemia cell morphology

An HL-60 cell suspension in PBS, containing approximately 1 × 10^5^ cells, was added into a cytospin chamber and centrifuged at 1000 rpm for 6 min at 4 °C. After air drying, the prepared cytospins were fixed in methanol for 15 min at room temperature. The cytospins were stained with the Hemacolor® Rapid Staining Kit according to the manufacturer’s instructions. The prepared cytospins were examined under 100x magnification in a blinded manner by the same observer, using an AXIO Scope.A1 microscope (Carl Zeiss, Germany). Based on the morphological features of differentiation, such as changes in cell size, alterations in the nuclear shape and the nucleus/cytoplasm ratio [29], the percentage of differentiated HL-60 cells was determined; 9000 leukemic cells were counted in each treatment group (3000 cells per slide).

#### Capacity to perform oxidative burst (NBT assay)

At the indicated time point, PMA at a final concentration of 100 nM was added to each well of a 96-well plate containing 100 μL of HL-60 cell suspension per well. The cells were incubated with PMA at 37 °C for 60 min, after which 10 μL of NBT solution (10 mg/mL) was added to each well and the cells were further incubated at 37 °C for 60 min. Following the incubation period, the cells were harvested by centrifugation and cytospin slides were prepared. A light microscope was used to identify NBT-positive cells that contained blue formazan deposits. A total of 200 cells on each slide were counted and the percentage of NBT-positive cells was calculated for each group.

#### Flow cytometric detection of CD11b expression

HL-60 cells were centrifuged, washed and resuspended in 100 μL of PBS. Then, 5 μL of either APC-conjugated CD11b antibody or APC-conjugated isotype control antibody was added to the suspension and the cells were incubated in the dark for 30 min at room temperature. After the incubation period, 400 μL of PBS was added to each tube and the cells were centrifuged. Finally, the cells were resuspended in 500 μL of PBS and analyzed in the FACSCalibur flow cytometer. 10,000 cells were examined in each sample and the percentage of CD11b-positive cells was determined using WinMDI 2.8 software.

### Assessment of apoptosis

#### Detection of apoptosis by light microscopy

Microscopic quantitative assessment of apoptosis was performed on cytospin slides prepared as described above and performed simultaneously with the analysis of cell differentiation. Based on the morphological features of apoptosis, such as cell shrinkage, chromatin condensation and nuclear fragmentation, the apoptotic index was calculated as the percentage of apoptotic HL-60 cells identified among 9000 leukemic cells (3000 cells per slide).

#### Annexin V-FITC/PI assay

Dual staining of HL-60 leukemic cells with fluorescein labelled annexin V (annexin V-FITC) and PI was performed using the FITC Annexin V Apoptosis Detection Kit I according to the manufacturer’s instructions. Briefly, the cells were washed twice with cold PBS and resuspended in 100 μl of the binding buffer. Then, 5 μL of annexin V-FITC and 5 μL of PI staining solution were added to the cell suspension and the cells were incubated in the dark for 15 min at room temperature. Following the incubation, 400 μL of the binding buffer was added to each tube. Cell samples were placed on ice away from light and FITC and PI fluorescence was immediately measured using the FACSCalibur flow cytometer. 10,000 cells were examined from each sample. The percentages of annexin V-FITC+/PI− cells (early apoptotic cells) and annexin V-FITC+/PI+ cells (late apoptotic and necrotic cells) were determined using WinMDI 2.8 software.

#### Caspase activity assay

A CellEvent™ Caspase 3/7 Green Flow Cytometry Assay Kit was used to detect activated caspases 3/7 in the leukemic cells. HL-60 cells were centrifuged and suspended in 0.5 mL of PBS. Next, 0.5 μL of CellEvent® Caspase-3/7 Green Detection Reagent was added and the cells were incubated for 30 min at 37 °C away from any light source. The reagent fluorescence was immediately measured using the FACSCalibur flow cytometer. 10,000 cells were examined from each sample and the percentage of cells with active caspase 3/7 was determined using WinMDI 2.8 software.

### Simultaneous detection of CD11b expression and annexin V binding

After harvesting, the cells were centrifuged, washed with PBS and suspended in 100 μl of annexin-V binding buffer. Then, the cells were double-stained with 5 μL of APC-conjugated anti-CD11b antibody and 5 μL of FITC-labeled annexin V for 30 min at room temperature. Following the incubation, the cells were washed with PBS and analyzed by flow cytometry. 10,000 cells were examined from each sample and the percentage of CD11b+/annexin V-FITC+ cells was determined using WinMDI 2.8 software.

### Western blot analysis of Bcl-2 expression

Hl-60 cells were washed with ice-cold PBS and lysed in ice-cold lysis buffer (50 mM Tris-HCl [pH 7.5] containing 100 mM NaCl, 0.5% sodium deoxycholate, 0.5% NP-40, 0.5% SDS and protease inhibitors), and the lysates were cleared by centrifugation at 15,000 g at 4 °C for 30 min. Protein content was determined via a protein assay using bovine serum albumin as a standard, after which western blots for Bcl-2 were performed. Proteins (30 μg from each experimental group) were separated by Mini-PROTEAN® TGX™ gels (Cat. No. 456–1093; BioRad), using SDS-PAGE (BioRad Mini-PROTEAN II Electrophoresis Cell). Proteins were transferred to PVDF membranes and incubated at 4 °C overnight with antibody diluted to 1:1000. Next, the membranes were incubated with a horseradish peroxidase-conjugated secondary antibody diluted to 1:1000. Signals were detected by chemiluminescence using the luminol reagent, visualized using a ChemiDoc-It Imaging System (UVP, LLC, Cambridge, UK) and quantified using ImageJ analysis software (US National Institute of Health, Bethesda, MD, USA). The blots were then stripped and probed for anti-β-actin.

### Statistical evaluation

The obtained results were confirmed by two or three independent experiments. All data are presented as the mean value ± the standard deviation (SD). Statistical analyses were performed using STATISTICA 10 (StatSoft, Kraków, Poland). Statistical significance for the data was evaluated by one-way analysis of variance (ANOVA) followed by Tukey’s honestly significant difference multiple range test. A *p* value *<*0.05 was considered statistically significant.

## Results

### Obatoclax affected HL-60 cell morphology

**Fig. 1:**
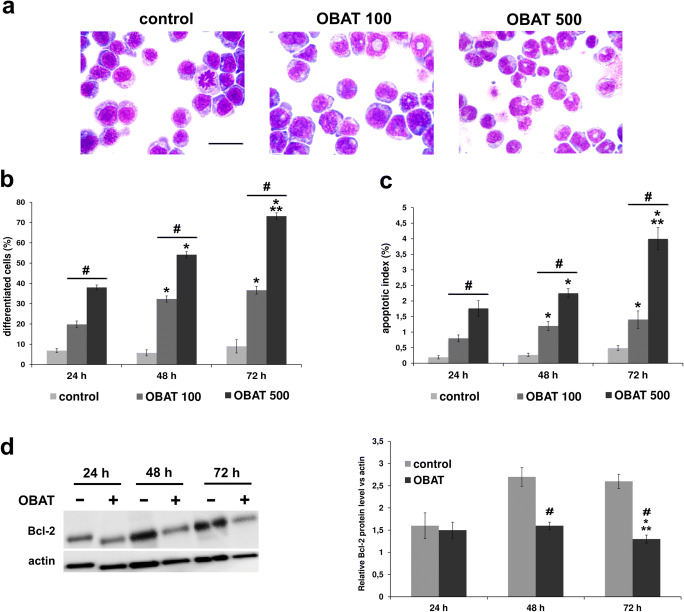
Effects of obatoclax on the morphology of HL-60 cells and levels of Bcl-2 protein in leukemic cells. **a** Representative images of HL-60 cells treated with 100 or 500 nM of obatoclax (OBAT) for 48 h and stained with Hemacolor demonstrating cells with characteristic features of differentiation (scale bar = 20 μm). Quantitative assessment of differentiated (**b**) and apoptotic (**c**) HL-60 cells performed under a light microscope. **d** Western blot analysis of Bcl-2 protein expression performed 24, 48 and 72 h after leukemic cells were exposed to OBAT at a concentration of 500 nM. Relative levels of Bcl-2 protein were quantified by densitometry and normalized to β-actin. The results are expressed as the mean values ± SD. Values significantly different (*p* ˂0.05) according to one-way ANOVA are designated by: * compared to 24 h, ** compared to 48 h, # compared to the corresponding control

The effects of OBAT on the morphology of HL-60 cells was studied over a time course of 72 h (Fig. 1a–c). After 24, 48 and 72 h, less than 10% of the untreated HL-60 cells were differentiated. Exposure of HL-60 cells to OBAT resulted in morphological changes characteristic of differentiation to the granulocytic lineage, such as reduced cytoplasmic basophilia, reduced nuclear/cytoplasmic ratio and the appearance of condensed and lobulated nuclei; all cells fulfilling this criteria were considered to be in the process of differentiation (Fig. 1a). Following 24 h of OBAT application at concentrations of 100 nM and 500 nM, 20% and 38% of HL-60 cells were differentiated, respectively. Exposure of HL-60 cells to OBAT for 48 and 72 h further increased the percentage of differentiated cells. The most pronounced morphological cell changes indicating differentiation were observed in the cells exposed to the higher concentration of OBAT (500 nM), which resulted in differentiation of 73% of the cells at the 72 h time point (Fig. 1b), at which time most of the HL-60 cells were observed to be in the metamyelocyte and band cell stages of differentiation. Simultaneously, apoptotic morphology occurring in HL-60 cells was noted to be both time- and dose-dependent. Compared to the untreated control cells, the most significant increase in the percentage of apoptotic cells was observed to occur in HL-60 cells exposed to OBAT at a concentration of 500 nM. The apoptotic index in the control group was 0.6% at the 72 h time point compared to 1.5% and 4% in the cells treated with 100 nM and 500 nM of OBAT, respectively (Fig. 1c).

### Obatoclax induced changes in the Bcl-2 protein level

In light of the key role of the Bcl-2 protein in hematopoietic cell differentiation and apoptosis, we measured Bcl-2 activation in HL-60 cells exposed to OBAT. Compared to the control group, leukemic cells exposed to OBAT exhibited a reduced level of Bcl-2 expression. The results of western blot analyses revealed 1.5- and 2-fold decreases in Bcl-2 protein levels in leukemic cells following 48 and 72 h of treatment with OBAT, respectively (Fig. 1d).

### The effect of obatoclax on the cell cycle

**Fig. 2:**
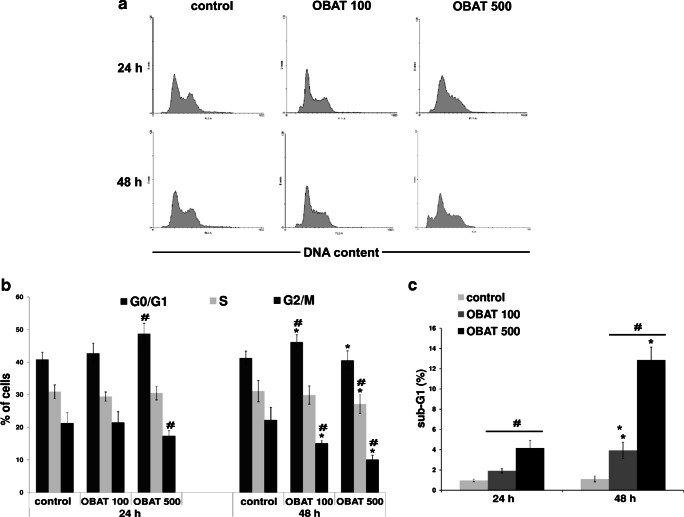
Effects of obatoclax on cell cycle distribution in HL-60 cells. **a** Representative histograms of the cell cycle in leukemic cells analyzed 24 and 48 h after exposure to 100 or 500 nM of obatoclax. **b** Changes in the percentage of HL-60 cells in specific phases of the cell cycle. **c** Changes in the percentage of the sub-G1 population. The results are expressed as the mean values ± SD. Values significantly different (*p* ˂0.05) according to one-way ANOVA are designated by: * compared to 48 h, # compared to the corresponding control

Previously, we demonstrated that OBAT treatment significantly decreased the number of HL-60 cells in a dose- and time-dependent manner [27]. Because myeloid differentiation is associated with a loss of proliferative capacity [35], in the present study we investigated the effect of obatoclax on cell cycle distribution. To accomplish this, HL-60 cells were stained with PI and flow cytometric analysis of DNA content was performed (Fig. 2a). Compared with the control values, 24 h of exposure to OBAT at a concentration of 500 nM resulted in a marked accumulation of cells in the G0/G1 phase accompanied with a significant reduction of cells in the G2/M phase of cell cycle. Furthermore, treatment of cells with 500 nM of OBAT for 48 h decreased the percentage of cells in the G0/G1 phase and further reduced the percentage of cells in the S and G2/M phases of the cell cycle. Simultaneously to these measurements, the percentage of the sub-G1 population was found to be markedly increased. Treatment with OBAT at a concentration of 100 nM caused G0/G1 cell cycle arrest and significantly increased the percentage of cells in the sub-G1 population at the 48 h time point (Fig. 2b, c).

### Obatoclax promoted differentiation of HL-60 cells

**Fig. 3:**
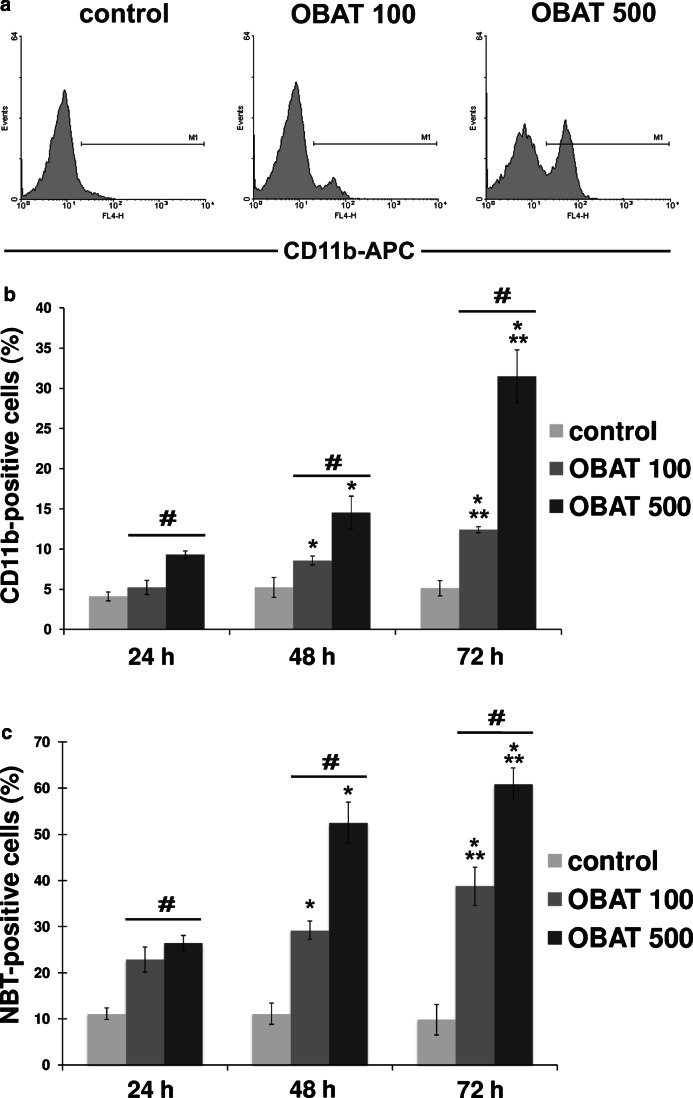
Effects of obatoclax on the differentiation of HL-60 cells evaluated 24, 48 and 72 h after treatment at concentrations of 100 nM and 500 nM. **a** Representative histograms of CD11b expression on HL-60 cells measured by flow cytometry 72 h after leukemic cell exposure to obatoclax. **b** The percentages of CD11b-positive HL-60 cells. **c** Changes in the percentages of cells performing oxidative burst (NBT-positive cells). The results are expressed as the mean values ± SD. Values significantly different (*p* ˂0.05) according to one-way ANOVA are designated by: * compared to 24 h, ** compared to 48 h, # compared to the corresponding control

To further verify the pro-differentiating effect of OBAT in HL-60 cells, we analyzed the cell surface expression of CD11b and determined the ability of these cells to produce superoxide and reduce NBT. As indicated in Fig. 3a, b, exposure of cells to OBAT for 24, 48 and 72 h increased the number of cells expressing the CD11b antigen compared to their respective controls. Approximately 4% of untreated HL-60 cells were CD11b-positive at each time point while, following OBAT application at concentrations of either 100 nM or 500 nM, 5% and 9% of HL-60 cells were found to express the CD11b antigen after 24 h, respectively. Longer incubations with OBAT increased the percentages of CD11b-positive cells. The highest percentage of CD11b-positive cells, 31%, was found in cells exposed to 500 nM of OBAT for 72 h (Fig. 3b). Furthermore, a marked time- and dose- dependent increase in the percentage of NBT-positive cells occurred as a result of OBAT treatment. The NBT reduction capability of HL-60 cells increased in a dose- and time- dependent manner following treatment with OBAT. As such, 72 h of exposure to OBAT at concentrations of 100 nM or 500 nM resulted in 38% and 60% of the cells being NBT-positive, respectively (Fig. 3c).

### Obatoclax enhanced apoptosis in HL-60 cells

**Fig. 4:**
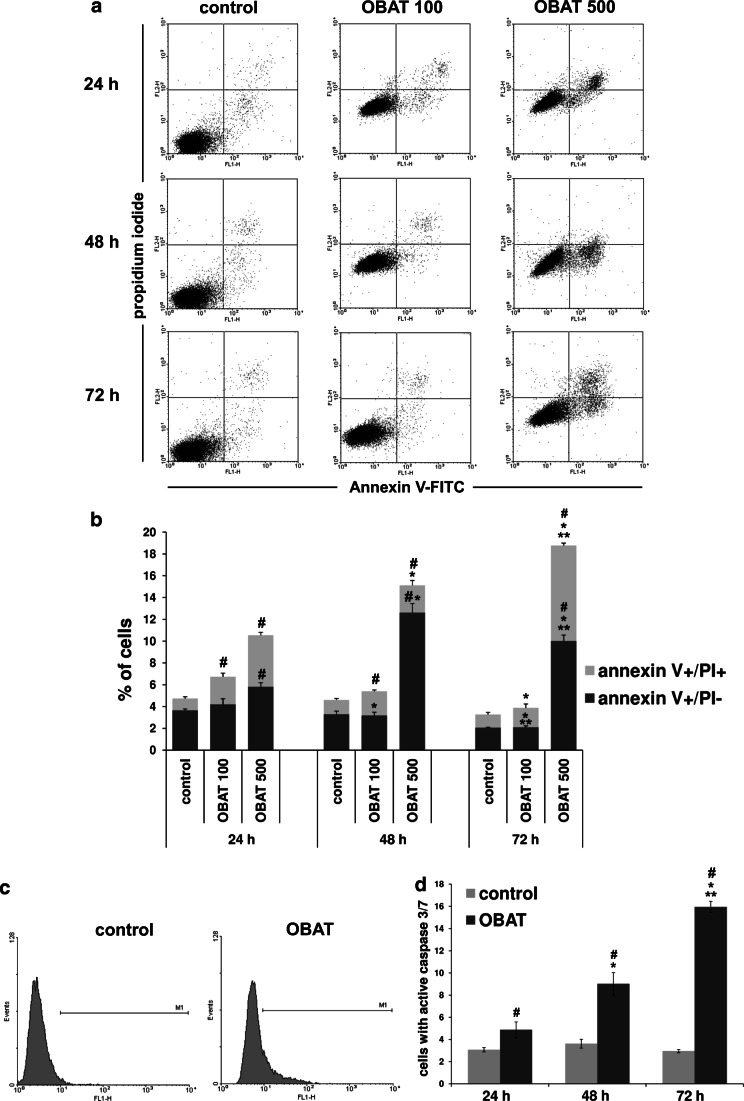
Effect of obatoclax on the induction of apoptosis in HL-60 cells. **a** Representative flow cytometry dot plots showing HL-60 cells stained with annexin V-FITC and propidium iodide (PI). **b** Changes in the percentages of early apoptotic cells (annexin V-FITC positive/PI negative) and late apoptotic and necrotic cells (annexin V-FITC positive/PI positive) were assessed 24, 48 and 72 h after leukemic cells were treated with obatoclax (OBAT) at concentrations of 100 nM and 500 nM. **c** Representative histograms of caspase 3/7 activation measured by flow cytometry 72 h after leukemic cell exposure to OBAT. **d** The percentage of HL-60 cells with active caspase 3/7 was determined 24, 48 and 72 h after cell exposure to 500 nM of OBAT. The results are expressed as the mean values ± SD. Values significantly different (*p* ˂0.05) according to one-way ANOVA are designated by: * compared to 24 h, ** compared to 48 h, # compared to the corresponding control

In order to confirm that OBAT significantly increases apoptosis of HL-60 cells, we tested the ability of OBAT to trigger phosphatidylserine externalization, plasma membrane disruption and caspase 3/7 activity in these cells. To achieve this, we used flow cytometry to assess the percentage of early apoptotic cells, as well as the percentage of HL-60 cells undergoing late apoptosis and necrosis following treatment (Fig. 4a, b). As shown in Fig. 4b, OBAT at a concentration of 100 nM significantly increased cell death following both 24 and 48 h of treatment. Treatment with OBAT at a concentration of 500 nM resulted in even more profound cell death, with the most significant induction of apoptosis and necrosis observed following treatment at this concentration for 72 h. Moreover, when comparing the values from HL-60 cells exposed to OBAT at the highest concentration (500 nM) for various time periods, a shift from early apoptosis to late apoptosis and necrosis was noted between the 48 and 72 h time points. Following 48 h of exposure to 500 nM of OBAT, 12% of HL-60 cells were annexin V-FITC+/PI- and 3% were annexin V-FITC+/PI+, whereas at the 72 h time point 9% of the cells were found to be in the early apoptotic stage and 10% were in the late apoptotic or necrotic stage (Fig. 4b). Accordingly, the activity of caspase 3/7 was significantly increased in the HL-60 cells treated with 500 nM of OBAT (Fig. 4c, d). The highest percentage of leukemic cells with active caspase 3/7 was found in the group of cells exposed to OBAT for 72 h (Fig. 4d).

### Differentiation of HL-60 cells was associated with the induction of apoptosis

**Fig. 5:**
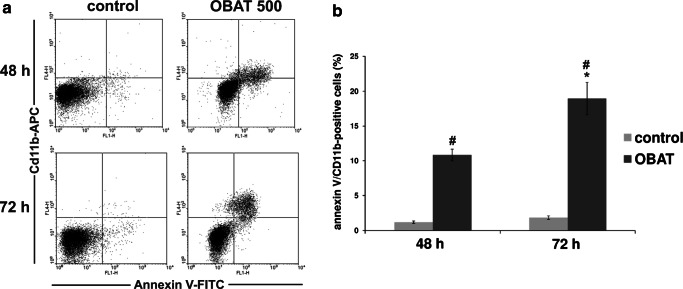
CD11b expression and annexin-V binding by obatoclax-treated HL-60 cells. Cells were exposed to 500 nM of obatoclax (OBAT) for 48 and 72 h and analyzed by flow cytometry. **a** Representative flow cytometry dot plots showing HL-60 cells stained with annexin V-FITC and CD11b. **b** Changes in the percentages of double positive cells for annexin V and CD11b. The results are expressed as the mean values ± SD. Values significantly different (*p* ˂0.05) according to one-way ANOVA are designated by: * compared to 48 h, # compared to the corresponding control

In order to determine whether the apoptosis induced by OBAT in HL-60 cells was associated with cell differentiation, simultaneous detection of CD11b expression and annexin V binding was performed in HL-60 cells exposed to OBAT for 48 and 72 h (Fig. 5a). Compared to the control group, treatment of HL-60 cells with OBAT resulted in a significant increase in the percentage of annexin V-FITC/CD11b-positive cells. The most pronounced effect was observed at the 72 h time point, in which 19% of cells were found to simultaneously exhibit phosphatidylserine externalization and CD11b expression (Fig. 5b). This observation suggests that, in a large portion of the OBAT-treated cells, differentiation resulted in apoptosis.

## Discussion

The recent development of Bcl-2 inhibitors has offered a novel therapeutic approach to treating cancer. As shown by previous studies, compounds such as obatoclax, ABT-737, ABT-199 and AT-101 exert potent pro-apoptotic activity, thus making them valuable weapons in the fight against cancer [36, 37]. Additional effects of Bcl-2 inhibitors on cancer cells beyond their role in triggering apoptosis have been demonstrated, including the induction of cell cycle arrest, the inhibition of cell proliferation and the induction of autophagy and necrotic cell death [20, 38, 39]. A better understanding of the mechanisms by which Bcl-2 antagonists exert their anticancer action is of key importance for their further preclinical and clinical development.

For the first time, our current study demonstrates the pro-differentiating activity of the pan-Bcl-2 inhibitor obatoclax in the HL-60 cell line, which is widely accepted as a valuable model for studying the regulatory mechanisms of hematopoietic cell differentiation and apoptosis [30]. The morphological and genetic characteristics of HL-60 cells are well known, thus making these cells a valid model for testing anti-leukemic compounds and investigating their mechanisms of action [29, 30, 40]. Our results indicate that treatment of HL-60 cells with OBAT induces dose- and time-dependent differentiation of these leukemic cells. Following treatment with OBAT, HL-60 cells acquired a phenotype characteristic of more mature myeloid cells, such as their specific morphology, the ability to perform oxidative burst and higher expression of CD11b, a myeloid differentiation marker. Together with the induction of myeloid maturation, OBAT treatment triggered dose- and time-dependent apoptosis in HL60 cells, as determined through morphological changes, increased percentages of annexin V-FITC-positive cells and elevated numbers of cells with active caspase 3/7. Differentiation and apoptosis in HL-60 cells was markedly increased by higher concentrations of OBAT, indicating that the pro-differentiating effect of OBAT is closely associated with its cytotoxicity. Previous studies have demonstrated that, during differentiation, AML cells undergo a number of marked changes in apoptosis-related genes that make them more susceptible to the apoptotic process [12, 40, 41]. The pro-apoptotic phenotype of these differentiated cells was shown to correlate with a decreased expression of anti-apoptotic proteins of the Bcl-2 family. The down-regulation of anti-apoptotic Bcl-2 proteins during myeloid differentiation has been well established [12, 42]. Decreased expression of Bcl-2 proteins appears to be part of the differentiation pathway and may serve to facilitate the apoptotic response [12]. Studies have shown that HL-60 cells induced to differentiate into neutrophils down-regulate Bcl-2 [43], an observation that is consistent with the findings from our study. We first noted suppression of Bcl-2 expression 48 h after HL-60 cells were exposed to OBAT, correlating with increased cell differentiation and apoptosis and suggesting that decreased Bcl-2 expression may be one of the events triggering the apoptosis in these cells. Of note, the pro-differentiating drug ATRA has been shown to repress Bcl-2 mRNA in differentiated AML cells [44, 45]. However, the differentiation of HL-60 cells exposed to OBAT could also partially occur through Bcl-2-independent mechanisms. Previous studies have shown that the over-expression of Bcl-2 in HL-60 cells suppresses apoptosis without producing any effects on the differentiation process [46]. Moreover, although many studies have shown down-regulation of anti-apoptotic Bcl-2 proteins following obatoclax treatment [19, 22], recent studies have indicated that obatoclax can also act through other cellular targets, such as p38/p21 [26] and mTOR [47] signaling pathways. Additional experiments are needed to firmly establish the role of Bcl-2 in obatoclax-induced differentiation of HL-60 cells.

Evident temporal changes in the percentages of differentiated and apoptotic HL-60 cells observed in the present study indicated that the restoration of apoptotic potential may be associated with progression of differentiation. To investigate this further, we co-stained HL-60 cells for annexin V and CD11b and observed that a large proportion of the differentiated cells were undergoing apoptosis; this was especially evident following longer durations of OBAT treatment (72 h). This observation is in agreement with previous findings showing that cultures of terminally differentiated myeloid leukemia cells contain a proportion of apoptotic cells [30, 48], which is especially noted after longer incubation periods of myeloid cells with differentiation inducers [46, 49]. However, it has also been reported that maturation of AML cells is not always necessary for triggering apoptosis [46], in fact differentiation and apoptosis can occur as separate events under different regulation in AML cells [50]. Therefore, it is noteworthy that differentiation of HL-60 cells following OBAT treatment may occur both simultaneously and independently of cell death, although further analysis is needed to clarify if this situation occurs in AML cells.

Previous in vitro studies have demonstrated that obatoclax’s anti-leukemic activity in AML cell lines and primary AML samples involves decreased cell proliferation [19]. Consistent with this data, the ability of OBAT to promote HL-60 maturation in the current study was shown to be coupled to cell cycle arrest in the G0/G1 phase. This cell cycle regulating property of obatoclax, via G1 phase arrest, has also been demonstrated in human colorectal [25, 51], esophageal [26] and thyroid [52, 53] cancer cells. As shown by Wei et al., exposure of thyroid cancer cells to obatoclax resulted in enrichment of cells in the G0/G1 phase accompanied with decreased cells in the S and G2/M phases in a concentration-dependent manner [53]. Cell cycle arrest induced by obatoclax was found to be correlated with reduced expression of cyclin D1 [51, 53]. Moreover, the delayed cell cycle of colorectal cancer cells was not influenced by over-expression of pro-survival Bcl-2 proteins [25]. These findings suggest that obatoclax’s effect on the cell cycle is Bcl-2 independent. Previous studies have demonstrated that the most pronounced effect Bcl-2 has on the cell cycle is to delay the progression from G0/G1 phases to S phase [54, 55]. Therefore, direct inhibition of Bcl-2 should facilitate G1/G0 to S phase transition instead of inducing G1/G0 phase arrest. This consideration indicates that non-canonical signaling pathways may be targeted by obatoclax. Moreover, the correlation between cell cycle arrest and AML cell differentiation following obatoclax treatment must be further established. In the present study, cell cycle arrest in the G0/G1 phase along with reduction of G2/M phase cell populations, was observed 24 h after HL-60 cell treatment with OBAT while a substantial increase in the percentage of differentiated cells was noted 48 h after treatment. Based on these results, it can be proposed that the cell cycle arrest induced by OBAT contributes to its pro-differentiation activity.

In conclusion, our study has shown for the first time that obatoclax, a small-molecule inhibitor of the Bcl-2 protein family, promotes maturation of HL-60 cells. This pro-differentiation effect was associated with the ability of obatoclax to induce cell cycle arrest and apoptosis in AML cells. Aside from obatoclax, it is worth noting that gossypol, another pan-Bcl-2 inhibitor, has also been shown to induce differentiation in leukemic cells [56]. Therefore, the anti-leukemic effects of Bcl-2 inhibitors may be more complex than thus far determined. It can be assumed that the anti-AML activity of obatoclax involves the induction of differentiation as well as apoptosis. However, further mechanistic studies will be required to establish which differentiation and cell death pathways are involved. Nevertheless, the pro-differentiation activity of obatoclax offers a new promising treatment approach for AML that merits further studies.

## Abbreviations

AML: acute myeloid leukemia
Annexin V-FITC: fluoresceinated annexin V
ATRA: all-*trans*-retinoic acid
Bcl-2: B cell lymphoma 2
Bcl-xL: B cell lymphoma extra-large
Bcl-w: Bcl-2-like protein 2
Bax: Bcl-2-associated X protein
Bak: Bcl-2-associated killer
BH: Bcl-2 homology domain
DMSO: dimethyl sulfoxide
Mcl-1: myeloid cell leukemia-1
NBT: nitro blue tetrazolium
OBAT: obatoclax
PI: propidium iodide
PMA: phorbol 12-myristate 13-acetate
